# *Scx*-Transduced Tendon-Derived Stem Cells (TDSCs) Promoted Better Tendon Repair Compared to Mock-Transduced Cells in a Rat Patellar Tendon Window Injury Model

**DOI:** 10.1371/journal.pone.0097453

**Published:** 2014-05-15

**Authors:** Chunlai Tan, Pauline Po Yee Lui, Yuk Wa Lee, Yin Mei Wong

**Affiliations:** 1 Department of Orthopaedics and Traumatology, Faculty of Medicine, The Chinese University of Hong Kong, Hong Kong SAR, China; 2 The Hong Kong Jockey Club Sports Medicine and Health Sciences Centre, Faculty of Medicine, The Chinese University of Hong Kong, Hong Kong SAR, China; 3 Headquarter, Hospital Authority, Hong Kong SAR, China; University of Rochester, United States of America

## Abstract

We hypothesized that the transplantation of *Scx*-transduced tendon-derived stem cells (TDSCs) promoted better tendon repair compared to the transplantation of mock-transduced cells. This study thus aimed to investigate the effect of *Scx* transduction on the expression of lineage markers in TDSCs and the effect of the resulting cell line in the promotion of tendon repair. Rat non-GFP or GFP-TDSCs were transduced with *Scx* or empty lentiviral vector (Mock) and selected by blasticidin. The mRNA expressions of *Scx* and different lineage markers were examined by qRT-PCR. The effect of the transplantation of GFP-TDSC-Scx on tendon repair was then tested in a rat unilateral patellar tendon window injury model. The transplantation of GFP-TDSC-Mock and scaffold-only served as controls. At week 2, 4 and 8 post-transplantation, the repaired patellar tendon was harvested for *ex vivo* fluorescent imaging, vivaCT imaging, histology, immunohistochemistry and biomechanical test. GFP-TDSC-Scx consistently showed higher expressions of most of tendon- and cartilage- related markers compared to the GFP-TDSC-Mock. However, the effect of *Scx* transduction on the expressions of bone-related markers was inconclusive. The transplanted GFP-TDSCs could be detected in the window wound at week 2 but not at week 4. Ectopic mineralization was detected in some samples at week 8 but there was no difference among different groups. The GFP-TDSC-Scx group only statistically significantly improved tendon repair histologically and biomechanically compared to the Scaffold-only group and the GFP-TDSC-Mock group at the early stage of tendon repair. There was significant higher expression of collagen type I in the window wound in the GFP-TDSC-Scx group compared to the other two groups at week 2. The transplantation of GFP-TDSC-Scx promoted healing at the early stage of tendon repair in a rat patellar tendon window injury model.

## Introduction

Scleraxis (Scx) is a basic helix-loop helix (bHLH) transcription factor which is present in tendon starting from the condensation stage and persists into adulthood [1]. Scx forms heterodimer with NFAT-C (Nuclear factor of activated T-cells, cytoplasmic) and directly regulates gene transcription of collagen type I (*Col1a1*), which encodes the most abundant extracellular matrix protein in tendons [2]. Scx knock-outs impair formation of load-bearing tendons [3]. Thus, Scx is a key regulator of tendon differentiation [3].

Tendon injury is common in various physical activities. While healed, the extracellular matrix composition of the repaired tendon is changed, with ectopic chondro-ossification in some tendon samples at the later stage of repair [4]. Erroneous cell differentiation to the non-tenocyte lineages during tendon repair might account for ectopic chondro-ossification in tendons after injury [5]–[6]. The biomechanical properties were compromised [7], which might account for the high re-tear rate of repaired tendon.

The transplantation of mesenchymal stem cells (MSCs) to the injury sites has been reported to promote tendon repair [8]–[10]. However, the transplantation of bone marrow-derived stem cells (BMSCs) at high concentration to the tendon defect was reported to induce ectopic bone formation [8], [11]–[12]. Tumor formation after transplantation of undifferentiated BMSCs was also reported under special circumstances [13]. Tendon-derived stem cells (TDSCs) are cells isolated from tendon tissues of various species and they form cell colonies, express stem cell-related markers and exhibit multi-lineage differentiation potential [14], [15]. They were shown to be different from tenocytes in a previous study [16]. Our group has shown that the transplantation of TDSCs histologically and biomechanically promoted tendon repair up to week 4 post-transplantation [17]. As tenocytes are the key machinery for the production of tendon matrix in tendon, we asked if increasing the tenogenic activity of TDSCs *in vitro* prior to transplantation would promote better tendon repair by enhancing the production of appropriate tendon matrix and reducing erroneous cell differentiation which might lead to ectopic chondro-ossification [18]. A previous study reported that lentiviral transduction of scleraxis (*Scx*) into an immortalized human BMSC line induced its tenogenic differentiation as indicated by the significantly higher collagen type I secretion, higher protein and mRNA expressions of tenomodulin (Tnmd), higher mRNA expression of decorin and lower mRNA expression of *Sox9* [19]. Only 2 tendon-related markers (Tnmd and collagen type I) were studied. The decrease in the multi-lineage differentiation potential, colony-forming ability and proliferative potential of the *Scx*-transduced immortalized BMSCs only demonstrated that the cells had a more restricted differentiation potential but this gives no conclusion on their specific lineage commitment. The transplantation of BMSCs overexpressing Scx [20], but not non-transduced BMSCs [21], was reported to promote tendon-bone junction healing in a rotator cuff healing rat model. However, the effect of TDSCs transduced with *Scx* on tendon repair has not been reported.

We hypothesized that the transplantation of *Scx*-transduced TDSCs promoted better tendon repair compared to the transplantation of mock-transduced cells. Therefore, in this study, we aimed to examine the effect of *Scx* transduction on the expression of more lineage markers in TDSCs. The effect of the resulting cell line on tendon repair was also evaluated in a rat patellar tendon window injury model.

## Materials and Methods

### Isolation and Culture of Rat TDSCs

All experiments were approved by the Animal Research Ethics Committee of the Chinese University of Hong Kong (10/023/GRF). All surgeries were performed under general anesthesia and all efforts were made to minimize suffering of animals. 4–6-week-old male outbred Green Fluorescent Protein (GFP) Sprague-Dawley (SD) rats (SD-Tg (CAG-EGFP) Cz-004Osb) and non-GFP SD rats, both weighting 150 to 220 g, were used for TDSC isolation as described previously [22] and shown in Supporting information S1. The clonogenicity and multi-lineage differentiation potential of the isolated cells were confirmed by standard assays (results not shown).

### Lentiviral Transduction of TDSCs

**Figure 1:**
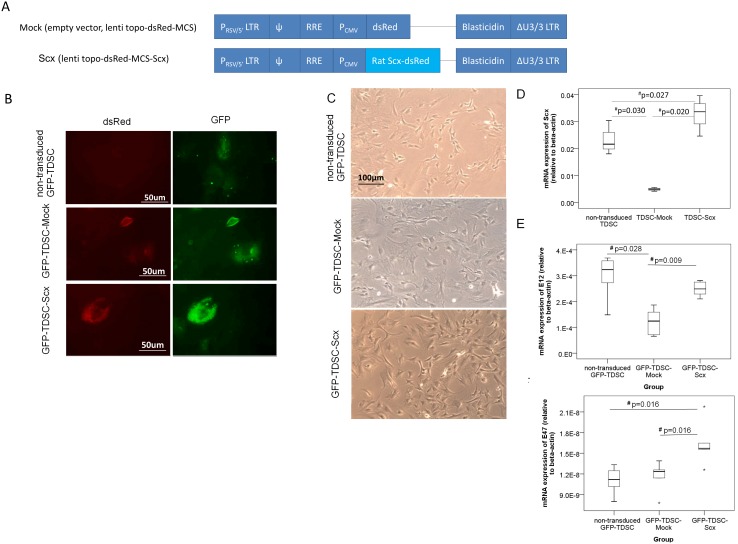
Transduction of *Scx* into TDSCs. (A) Schematic diagram of the lenti topo-dsRed-MCS-Scx construct and the Mock construct; **P_RSV/5′_LTR:** long terminal repeat; **ψ:** HIV-1 packaging signal; **RRE:** HIV-1 Rev responsive element; **P_CMV_:** CMV promoter; **Rat Scx:** rat scleraxis gene; **dsRed:** red fluorescent protein; **ΔU3/3/LTR:** long terminal repeat; (B) Fluorescence of dsRed and GFP in non-transduced GFP-TDSC, GFP-TDSC-Mock and GFP-TDSC-Scx; Scale bar: 50 µm; (C) Cell morphology under light microscopy; Scale bar: 100 µm; (D) Boxplot showing *Scx* expression in TDSCs with or without lentiviral transduction of *Scx*. n = 3 different cell sources (1 TDSC and 2 GFP-TDSC sources); (E–F) Boxplots showing the mRNA expression of *Tcf3* (E12) (E) and *Tcf3* (E47) (F) in non-transduced GFP-TDSC, GFP-TDSC-Mock and GFP-TDSC-Scx. Note that non-transduced GFP-TDSC has lower cumulative population doublings compared to GFP-TDSC-Mock and GFP-TDSC-Scx while at the same passage. n = 5 per group; “*” represents extreme value of the dataset. #p≤0.05 in post-hoc comparison. Our results showed that *Scx* was successfully transduced into TDSCs as indicated by the red fluorescence of dsRed and the significantly higher mRNA expression of *Scx*. There was no observable change in cell morphology after Mock or *Scx* transduction. The mRNA expressions of E12 and E47, E-protein binding partners of Scx, were also significantly increased after *Scx* transduction. Note that the mRNA expression of *Scx* in the TDSC-Mock group was significantly lower compared to that in the non-transduced TDSC group, probably due to the higher numbers of cumulative population doublings of the transduced TDSCs compared to that in the non-transduced TDSCs at the same cell passage as over 85% of cells were killed during blasticidin selection.

A lentiviral vector for increasing the expression of Scx in TDSCs was constructed (Figure 1A). Briefly, the coding sequence of *Scx* gene (NM_001130508, 630 bp) was amplified from the cDNA of non-GFP rat TDSCs at passage 1 (P1) by RT-PCR using specific primers. The *Scx* gene was then cloned into the lentiviral vector, lenti topo-dsRed-MCS. A lentiviral vector without the *Scx* gene served as the control (Mock). The lentiviral vector was then transformed and packaged in 293FT cells. The lentiviral particles collected were then used for the infection of TDSCs isolated from one non-GFP SD rat and 2 GFP SD rats at passage 1–2 (P1–2). The transduced cells were then selected by 10 µg/ml blasticidin (InvivoGen, San Diego, USA) for 8 days that over 85% of the cells died during the selection. The remaining cells were then cultured until confluence and sub-cultured in complete culture medium containing low glucose Dulbecco’s Modified Eagle Medium (LG-DMEM), 10% FBS, 50 µg/ml penicillin, 50 µg/ml streptomycin, 100 µg/ml neomycin (all from Invitrogen, Carlsbad, CA, USA) and 2 µg/ml blasticidin. There were three TDSC-Scx lines, one with TDSCs isolated from a non-GFP SD rat and the other two with TDSCs isolated from two different GFP rats. Each TDSC-Scx line was compared to its corresponding TDSC-Mock line and non-transduced TDSC line (not an appropriate control as described below) with TDSCs isolated from the same rat. The successful introduction of lentiviral vector into TDSCs isolated from GFP rats (see result section) and non-GFP rat (results not shown) was confirmed by dsRed expression. The GFP SD rats have the same genetic background as the non-GFP SD rats, except for the expression of GFP. Hence we do not expect any differences in the results obtained with TDSCs isolated from GFP and non-GFP animals. The non-GFP TDSC-Scx line was generated for other on-going experiments in our laboratory where the GFP label in GFP-TDSCs was found to interfere with the assessments by fluorescent means.

To confirm the successfulness of *Scx* transduction into TDSCs, non-transduced TDSC, TDSC-Mock and TDSC-Scx of 3 different cell sources at P5–6 were seeded at 10^4^ cells/cm^2^ in 10 cm culture dishes until confluence. The mRNA expression of *Scx* was then examined by qRT-PCR. The mRNA expressions of *Tcf3* (E12) and *Tcf3* (E47), both binding partners of Scx to the E-box required for gene transcription, in non-transduced GFP-TDSC, GFP-TDSC-Mock and GFP-TDSC-Scx at P4 were also measured. The mRNA expressions of E12 and E47 were examined to ensure that the function of Scx would not be limited by the availability of E12 and E47. To measure the mRNA expressions of lineage markers, non-transduced GFP-TDSC, GFP-TDSC-Mock and GFP-TDSC-Scx at P4–5 were seeded at 10^4^ cells/cm^2^ until confluence. The mRNA expressions of tendon-related markers (scleraxis (*Scx*), thrombospondin-4 (*Thbs4*), tenascin C (*Tnc*), collagen type I (*Col1a1*), ephrin type-A receptor 4 (*Epha4*), eyes absent homolog 1 (*Eya1*), SIX homeobox 1 (*Six1*), SIX homeobox 2 (*Six2*), elastin (*Eln*), tenomodulin (*Tnmd*)), cartilage-related markers (aggrecan (*Acan*), biglycan (*Bgn*), collagen type II (*Col2a1*), *Sox9*, collagen type X (*Col10a1*)) and bone-related markers (Runx2 (*Runx2*), osteopontin (*Spp1*), osteocalcin (*Bglap*)) were then examined by qRT-PCR. Non-transduced GFP-TDSC, while included in the analysis, was not a valid control because GFP-TDSC-Scx and GFP-TDSC-Mock, while at the same passage as the non-transduced GFP-TDSC, underwent more population doublings due to blasticidin selection. Our previous study showed that there was loss of multi-lineage differentiation potential of TDSCs with *in vitro* passaging [23]. Indeed, we observed lower expressions of most of the lineage markers in the GFP-TDSC-Mock compared to those in the non-transduced GFP-TDSC (see result section). The mRNA expression of *Scx* in the non-transduced TDSC, TDSC-Mock and TDSC-Scx was the result of 3 independent cell sources. GFP-TDSC-Scx and its corresponding GFP-TDSC-Mock and non-transduced GFP-TDSC lines from the two GFP rats were then used for the subsequent mRNA analysis of lineage markers. One GFP-TDSC-Scx and its corresponding GFP-TDSC-Mock line with GFP-TDSCs isolated from the same GFP rat were used for transplantation in the animal model.

### Expression of dsRed

Non-transduced GFP-TDSC, GFP-TDSC-Mock and GFP-TDSC-Scx, with TDSCs isolated from the same GFP rat, at P5 were seeded at 6000 cells/cm^2^ on coverslips placed in 6-well-plates at 37°C, 5% CO_2_ overnight. The fluorescence of dsRed and GFP was examined using a fluorescent microscope equipped with a UV laser (ZEISS AxioPlan 2, Carl Zeiss MicroImaging LLC, Jena, Gemany). There were 3 replicates in each group.

### Quantitative Real Time RT-PCR (qRT-PCR)

The procedures were well-established and described previously [22]. The specific primers for *Scx*, *Thbs4*, *Tnc*, *Col1a1*, *Epha4*, *Eya1*, *Six1*, *Six2*, *Eln*, *Tnmd*, *Acan*, *Sox9*, *Col2a1*, *Bgn*, *Col10a1*, *Runx2*, *Spp1*, *Bglap*, *Tcf3* (E12), *Tcf3* (E47) or *β-actin* were shown in Table 1. The expression of target gene was normalized to that of *β-actin* gene. Except *Eya1* which there were 4–5 replicates in each group, there were 5–6 replicates in each group for the other target genes.

**Table 1:** Primer sequences and condition for qRT-PCR.

Gene	Primer nucleotidesequence	Product size (bp)	Annealingtemperature (°C)	Accession no
*β-actin*	5′-ATC GTG GGC CGC CCT AGG CA-3′ (forward)	243	52	NM_031144
	5′-TGG CCT TAG GGT TCA GAG GGG-3′ (reverse)			
*Tcf3* (E12)	5′-AAA CTG CTC ATC CTG CAC CA-3′ (forward)	127	58	NM_133524.2
	5′-CCA CGC CAG ATA CCT TCT CC-3′ (reverse)			
*Tcf3* (E47)	5′-AGG ACG AGG AGG TCA CAT CA-3′(forward)	123	58	NM_001035237.1
	5′-GTC CCT CAG GTC CTT CTC CT-3′ (reverse)			
*Scx*	5′-AAC ACG GCC TTC ACT GCG CTG-3′ (forward)	102	58	NM_001130508.1
	5′-CAG TAG CAC GTT GCC CAG GTG-3′ (reverse)			
*Thbs4*	5′-CCA CCT GCT CCG CTC ACT GC -3′ (forward)	263	60	NM23637.1
	5′-GTC AGG ACT GGC TGC AGG GC -3′ (reverse)			
*Tnc*	5′-CAG AAG CTG AAC CGG AAG TTG-3′ (forward)	278	55	NM_053861.1
	5′-GGC TGT TGT TGC TAT GGC ACT-3′ (reverse)			
*Col1a1*	5′-CAT CGG TGG TAC TAA C-3′ (forward)	238	50	NM_053356.1
	5′-CTG GAT CAT ATT GCA CA-3′ (reverse)			
*Epha4*	5′- GGA TGT GGG TGC TTG CAT CG-3′ (forward)	112	58	NM_001162411.1
	5′- GGT ATC AGC CCC AGT GAT GG-3′ (reverse)			
*Eya1*	5′- GAT CCG CCT ACT TCT GTT TC-3′ (forward)	182	60	XM_002729455.2
	5′- GGA ACA GAT GGC TTT CCT GC-3′ (reverse)			
*Six1*	5′-GGA GAA GTC TCG GGG CGT G-3′ (forward)	180	60	NM_053759.1
	5′-TTT TCG GTG TTC TCC CTT TCC T-3′ (reverse)			
*Six2*	5′-CTC ACC ACC ACG CAG GTC AG-3′ (forward)	169	60	NM_001191908.1
	5′-CGT CCT CGG AAC TGC CTA GC-3′ (reverse)			
*Eln*	5′-AAA GTT CCT GGT GTC GGT CTT CCA-3′ (forward)	528	60	NM_012722.1
	5′-AGC AGC TCC ATA CTT AGC AGC CTT-3′ (reverse)			
*Tnmd*	5′-GTG GTC CCA CAA GTG AAG GT-3′ (forward)	60	52	NM_022290.1
	5′-GTC TTC CTC GCT TGC TTG TC-3′ (reverse)			
*Acan*	5′-CTT GGG CAG AAG AAA GAT CG-3′ (forward)	159	58	NM_022190.1
	5′-GTG CTT GTA GGT GTT GGG GT-3′ (reverse)			
*Bgn*	5′-TCT ACA TCT CCA AGA ACC ACC TGG-3′ (forward)	514	55	NM_017087.1
	5′-TTG GTG ATG TTG TTG GAG TGC AGA-3′ (reverse)			
*Col2a1*	5′-ATG ACA ATC TGG CTC CCA ACA CTG C-3′ (forward)	364	55	BT007205
	5′-GAC CGG CCC TAT GTC CAC ACC GAA T-3′ (reverse)			
*Sox9*	5′-AGA GCG TTG CTC GGA ACT GT-3′ (forward)	67	55	NM_017087.1
	5′-TCC TGG ACC GAA ACT GGT AAA-3′ (reverse)			
*Col10a1*	5′-TGG GTA GGC CTG TAT AAG AAT GG-3′ (forward)	209	58	XM_001053056.4
	5′- ATG GGA GCC ACT AGG AAT CCT GA-3′ (reverse)			
*Runx2*	5′-CCG ATG GGA CCG TGG TT-3′ (forward)	75	58	NM_001278484.1
	5′-CAG CAG AGG CAT TTC GTA GCT-3′ (reverse)			
*Spp1*	5′-TCC AAG GAG TAT AAG CAG CGG GCC A-3′ (forward)	199	60	NM_23637.1
	5′-CTC TTA GGG TCT AGG ACT AGC TTC T-3′ (reverse)			
*Bglap*	5′-GAG CTG CCC TGC ACT GGG TG-3′ (forward)	263	55	NM_012881.2
	5′-TGG CCC CAG ACC TCT TCC CG-3′ (reverse)			

### Transplantation of GFP-TDSC-Scx in a Patellar Tendon Window Injury Model

One-hundred-and-twenty-two outbred non-GFP SD male rats (7–8 weeks, body weight of 250–320 g) were used in this study, of which 104 rats were used for surgery and the other 18 rats were used for the measurement of biomechanical properties of the central portion of intact tendon. The surgical procedures and the TDSC transplantation protocol were well-established [17]. To create the tendon defect, the central portion of the patellar tendon (∼1 mm in width) was removed from the distal apex of the patella to the insertion of the tibial tuberosity without damaging the fibrocartilage zone using three stacked sharp blades with the middle blade moved up to create a gap between the two outer blades. The operated rats were divided into 3 groups: (a) fibrin glue-only group (Scaffold-only group) (n = 36); (b) GFP-TDSC-Mock in fibrin glue group (GFP-TDSC-Mock group) (n = 33) and (c) GFP-TDSC-Scx in fibrin glue group (GFP-TDSC-Scx group) (n = 35). Our published results showed that TDSCs have low immunogenicity and exhibited weak immuno-reactions from hosts after transplantation and hence were suitable for allogeneic transplantation [24], [25].

To prepare the GFP-TDSC-fibrin constructs for transplantation, 60 µl of 1.6×10^7^ cells were mixed with 370 µl of fibrin glue solution A and then 370 µl of fibrin glue solution B (Beriplast(R) P Cmbi-Set, CSL Behring) [17], [21], [26], [27]. This was the highest possible cell concentration that we could suspend the cells in the fibrin glue solution. Eight-hundred microliters of the mixture were added to a 35 mm culture dish (8 cm^2^) and allowed to gel for 5 minutes. TDSC-fibrin constructs of dimension 1 mm (thickness)×10 mm (length)×2 mm (width) were then cut from the dish for transplantation. Each construct thus contained 4×10^5^ cells in 20 µl of fibrin glue. Constructs prepared in the same way without cells were used for transplantation in the Scaffold-only group.

The fibrin glue construct was placed and tight-fitted in the tendon defect with the length and width of the construct fitted into the length and width of the wound, respectively. The wound was closed in layers. The animals were allowed to have free-cage activity until euthanasia. The tracking of the transplanted GFP cells using *ex vivo* fluorescent imaging at the time of sample harvest showed that the construct remained in place. At week 2, 4 and 8, one batch of samples (n = 54) was harvested for *ex vivo* fluorescent imaging of GFP signal (n = 6 per group per time point), followed immediately by histology and immunohistochemistry (both n = 6 per group per time point). vivaCT imaging of ectopic mineralization in tendon was additionally performed prior to histology for the same batch of samples harvested at week 8 (n = 6 per group for the GFP-TDSC-Mock group and GFP-TDSC-Scx group; n = 3 for the Scaffold-only group). Another batch of samples (n = 50) was harvested at week 4 and week 8 for biomechanical test (n = 10 per group for week 4; n = 8, 5, 7 for the Scaffold-only group, GFP-TDSC-Mock group and GFP-TDSC-Scx group, respectively, for week 8).

### *Ex vivo* Fluorescence Imaging

The tracking of the transplanted cells in the tendon defect at the time of sample harvest was done by the IVIS 200 imaging system (Xenogen, Alameda, CA, USA) [17]. The harvested patellar tendons were scanned and images with pseudocolor indicating the fluorescent intensity of GFP were obtained (red least and yellow most intense).

### vivaCT Imaging

Ectopic mineralization in tendons was examined using a cone-beam vivaCT system (VivaCT40, Scanco Medical AG, Bassersdorf, Switzerland) according to our protocol [28]. The samples were scanned. The images were then 3D reconstructed and aligned after thresholding using the built-in software.

### Histology

The healing of the window wound was examined by histology and scoring according to our established protocol [4]. One coronal section in the middle of each patellar tendon was selected, stained with haematoxylin and eosin and examined under light microscopy (DMRXA2, Leica Microsystems Wetzlar GmbH, Germany). Collagen fiber alignment was examined under polarized light using the same microscope. References were made to the adjacent normal tendon tissue and normal patellar tendon in evaluating the healing outcomes.

The histological slides were evaluated using a further modified scoring system reported previously [29] which was modified based on the Movin score [30]. Nine parameters – (1) fiber arrangement; (2) cellularity; (3) cell alignment; (4) cell rounding; (5) vascularity; (6) fiber structure; (7) hyaline degeneration; (8) inflammation and (9) ossification were evaluated. These parameters were quantified using a 0–3 scale, with 0 being normal and 3 being maximally abnormal. The total score was calculated by summing the scores of the nine parameters. Vascularity was defined as the number of vascular features which was shown in another study to express the pericyte marker CD146 [31]. Hyaline degeneration was defined as the percentage of area with chondrocyte-like cells or ossification or degeneration which was confirmed in a previous study by immunohistochemical staining [28]. The whole window wound was assessed and scored at 200x magnification under the microscope. One slide in the middle of each patellar tendon was used for histological evaluation by two independent investigators. The histological scoring system was shown in a blind evaluation to have significantly high intra-rater reliability with intra-class correlation (ICC) of 0.840–1.000 (all p≤0.001) and significantly high inter-rater reliability with ICC of 0.818–1.000 (all p≤0.001) for all parameters. Similar conclusion was reached by both investigators and the scores of one investigator were presented. There were 6 slides (i.e. 6 tendons) in each group at each time point for scoring. The score of each parameter and the total score for each group at each time point were presented as median and range. Representative histological images matching the conclusion of histological scoring were presented.

### Biomechanical Test

The patellar-patellar tendon-tibia composite was first isolated. The healing tissue in the window wound connected to the bony ends was then isolated by excising the medial and lateral intact tendon using three stacked blades similar to the creation of tendon defect. While it was more difficult to identify the margins at the later time points, we could still differentiate the healing tissue from the intact tissue by naked eye. Any sample that might possibly be damaged during sample preparation was discarded. The composite was fixed on a custom-made testing jig with two clamps. The whole construct was then mounted onto the Hounsfield H25KS mechanical testing machine under tension (Tinius Olsen Ltd, Salfords, UK). After preconditioning, the pull to failure test was performed at a testing speed of 40 mm/min using a 50 N or 10 N load cell. The load-displacement curve and the failure mode of the healing tendon tissue were recorded. The ultimate stress (N/mm^2^) was calculated as the ultimate load divided by the averaged cross-sectional area of tendon estimated from the ultrasound images taken prior to the biomechanical test. The Young’s moduli (N/mm^2^) were calculated as the linear slope of the stress-strain curve at the step of 0.2 mm extension and the maximum slope was taken as the maximum Young’s modulus. There were 10 samples in each group at week 4. There were 8, 5 and 7 samples in the Scaffold-only group, GFP-TDSC-Mock group and GFP-TDSC-Scx group, respectively, at week 8. Two samples at week 4 (one in the GFP-TDSC-Mock group and one sample in the GFP-TDSC-Scx group) could not be used for the calculation of the ultimate stress and the maximum Young’s modulus as we forgot to set zero prior to performing the pull to failure test for one sample and we had problem with ultrasound imaging for the other sample. However, the failure modes of both samples were successfully recorded. The ultimate stress and the maximum Young’s modulus of the central portion of intact patellar tendons created using three stacked blades (n = 9 and at week 4 and week 8, respectively) were also measured and reported.

### Ultrasound Imaging

The cross-sectional area of healing tissue was measured using an animal ultrasound system (Vevo-770, VisualSonics, Toronto, Ontario, Canada) [17]. The 3D image of healing tissue was scanned. The tissue volume and length were measured by the system software. The average cross-sectional area was calculated as the tissue volume divided by the tissue length. Ultrasound imaging allows very accurate measurement of cross-sectional area of tendon to 0.01 mm and was done to adjust for the variability in cutting due to differences in tissue quality at the interfaces in different groups.

### Immunohistochemistry and Image Analysis

The expression of collagen type I in the patellar tendon window wound was examined by immunohistochemistry as described previously [4]. Briefly, after removal of paraffin and rehydration, endogenous peroxidase activity was quenched with 3% hydrogen peroxide for 20 minutes at room temperature. Antigen retrieval was then performed with citrate buffer at 65°C for 20 minutes. After blocking with 1% BSA and 5% donkey serum, anti-COL1A1 antibodies (1∶100; sc-8784, Santa Cruz Biotechnology, Inc., Texas, USA) were added to the sections in a humid chamber at 4°C overnight. The sections were then rinsed and treated with donkey anti-goat-HRP antibodies (1∶100, sc-2020, Santa Cruz Biotechnology, Inc., Texas, USA) for an hour at room temperature and rinsed. The immunopositive signal was visualized by adding DAB Quanto Chromogen (Lab Vision, Fremont, CA, USA) for 5 minutes at room temperature. The Image Pro Plus software (MediaCybernetics, Bethesda, MD, USA) was used for image analysis. One photomicrograph from one section of each tendon sample, covering the whole tendon mid-substance, was taken under the same camera setting. The window wound was selected as the region of interest (ROI) manually. Segmentation of the image was then performed with the “Select Colors” command of the software with hue: 1–40, saturation: 1–255 and intensity: 1–180 to select the immunopositive signal. The integrated optical density (in arbitrary unit) of the ROI was then measured using the “Count” command of the software. The area of ROI was also measured. The IOD/µm^2^ for each tendon sample was reported. There were 5–6 samples in each group at each time point. The representative images matching the conclusion of the semi-quantitative image analysis were selected for presentation.

### Data Analysis

The mRNA, biomechanical test and immunohistochemical data were shown in boxplots. Histological scores were presented as median and range. The comparison of 2 independent groups was done by Mann-Whitney U test. The comparison of more than 2 independent groups was done by Kruskal-Wallis test followed by post-hoc pairwise comparison using the Mann-Whitney U test. For the analysis of *Scx* expression in cell lines prepared from 3 different cell sources, the expression of *Scx* in the TDSC-Scx group was compared to its corresponding TDSC-Mock and non-transduced TDSC groups by repeated measures of ANOVA followed by post-hoc pair-wise comparison using the LSD method. All the data analysis was done using the SPSS analysis software (SPSS Inc, Chicago, IL, version 16.0). *p*≤0.05 was regarded as statistically significant.

## Result

### Expression of *Scx* in TDSCs after Lentiviral Transduction of *Scx*

The successful transduction of *Scx* into TDSCs was confirmed by the fluorescence of dsRed and the mRNA expression of *Scx*. The transduced GFP cells showed red fluorescence of dsRed (Figure 1B). Similar results were observed for the transduced non-GFP cells (results not shown). There was no observable change in cell morphology in the GFP-TDSCs after Mock or *Scx* transduction (Figure 1C). The successful transduction of *Scx* into TDSCs was confirmed by the significantly higher mRNA expression of *Scx* in the GFP-TDSC-Scx group compared to that in the non-transduced TDSC (post-hoc p = 0.027) and TDSC-Mock groups (post-hoc p = 0.020) (Figure 1D). We did not observe any obvious difference in *Scx* expression between the *Scx*-transduced GFP and non-GFP cell lines. The mRNA level of *Scx* in the *Scx*-transduced non-GFP cell line was between the two Scx-transduced GFP cell lines. Both the mRNA expressions of *Tcf3* (E12) (post-hoc p = 0.009) (Figure 1E) and *Tcf3* (E47) (post-hoc p = 0.016) (Figure 1F) were significantly higher in the GFP-TDSC-Scx group compared to those in the GFP-TDSC-Mock group, suggesting that the functions of Scx were unlikely to be limited by the availability of its E-protein binding partners after transduction. There was significantly lower expression of *Scx* in the TDSC-Mock group compared to that in the non-transduced TDSC group (post-hoc p = 0.030) (Figure 1D). The mRNA expression of *E12* (post-hoc p = 0.028) (Figure 1E), but not *E47* (Figure 1F), was also significantly lower in the GFP-TDSC-Mock group compared to that in the non-transduced GFP-TDSC group.

### mRNA Expressions of Lineage Markers

**Figure 2:**
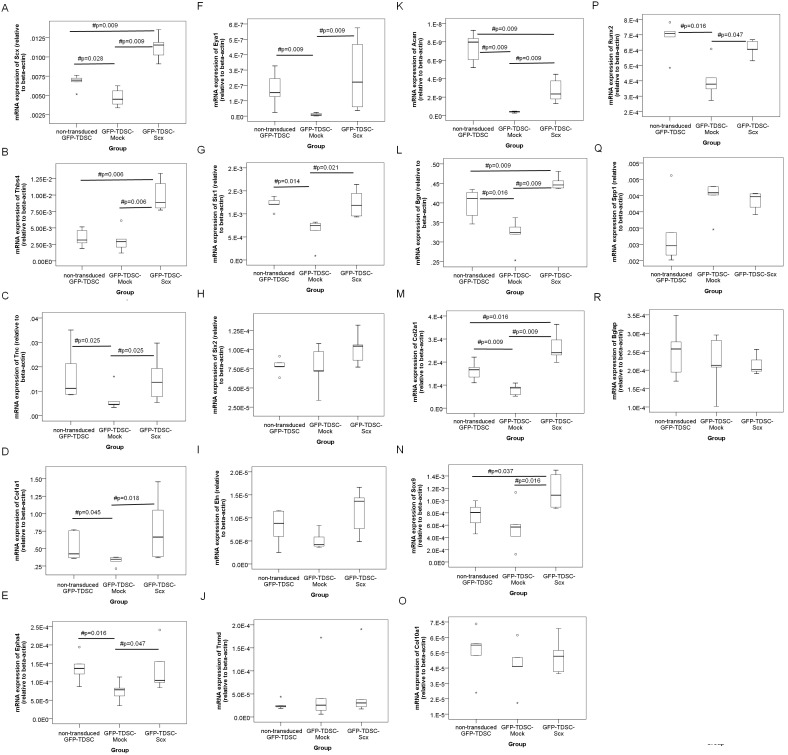
mRNA expressions of lineage markers after transduction of *Scx* into TDSCs. Boxplots showing the mRNA expressions of (A) *Scx*, (B) *Thbs4*, (C) *Tnc*, (D) *Col1a1*, (E) *Epha4*, (F) *Eya1*, (G) *Six1*, (H) *Six2*, (I) *Eln*, (J) *Tnmd*, (K) *Acan*, (L) *Bgn*, (M) *Col2a1*, (N) *Sox9*, (O) *Col10a1*, (P) *Runx2*, (Q) *Spp1* and (R) *Bglap* in the non-transduced GFP-TDSC, GFP-TDSC-Mock and GFP-TDSC-Scx. Note that the non-transduced GFP-TDSC has undergone lower numbers of cumulative population doublings compared to the GFP-TDSC-Mock and GFP-TDSC-Scx at the same passage and hence is not a valid control. n = 4–6 per group; #post-hoc p≤0.05; “o” and “*” represent outlier and extreme value of the dataset, respectively. The expressions of most of the tendon-related markers (*Thbs4*, *Tnc*, *Col1a1*, *Epha4*, *Eya1*, *Six1*) and cartilage-related markers (*Acan*, *Bgn*, *Col2a1*, *Sox9*) were significantly higher in the GFP-TDSC-Scx compared to those in the GFP-TDSC-Mock. Only the bone-related marker, *Runx2*, significantly increased in the GFP-TDSC-Scx compared to that in the GFP-TDSC-Mock.

We next analyzed the mRNA expressions of tendon-, cartilage- and bone- related markers in the GFP-TDSCs after *Scx* transduction. Similar to the results in Figure 1D, there was significant higher expression of *Scx* in the GFP-TDSC-Scx compared to that in the non-transduced GFP-TDSC (post-hoc p = 0.009) and GFP-TDSC-Mock (post-hoc p = 0.009) (Figure 2A). The expressions of most markers including *Scx* (post-hoc p = 0.028) (Figure 2A), *Tnc* (post-hoc p = 0.025) (Figure 2C), *Col1a1* (post-hoc p = 0.045) (Figure 2D), *Epha4* (post-hoc p = 0.016) (Figure 2E), *Eya1* (post-hoc p = 0.009) (Figure 2F), *Six1* (post-hoc p = 0.014) (Figure 2G), *Acan* (post-hoc p = 0.009) (Figure 2K), *Bgn* (post-hoc p = 0.016) (Figure 2L), *Col2a1* (post-hoc p = 0.009) (Figure 2M) and *Runx2* (p = 0.016) (Figure 2P) were significantly lower in the GFP-TDSC-Mock compared to those in the non-transduced GFP-TDSC. There were significant higher expressions of tendon-related markers including *Thbs4* (post-hoc p = 0.006) (Figure 2B), *Tnc* (post-hoc p = 0.025) (Figure 2C), *Col1a1* (post-hoc p = 0.018) (Figure 2D), *Epha4* (post-hoc p = 0.047) (Figure 2E), *Eya1* (post-hoc p = 0.009) (Figure 2F) and *Six1* (post-hoc p = 0.021) (Figure 2G) but not *Six2* (Figure 2H), *Eln* (Figure 2I) and *Tnmd* (Figure 2J) in the GFP-TDSC-Scx compared to those in the GFP-TDSC-Mock. There were also significant higher expressions of cartilage-related markers including *Acan* (post-hoc p = 0.009) (Figure 2K), *Bgn* (post-hoc p = 0.009) (Figure 2L), *Col2a1* (post-hoc p = 0.009) (Figure 2M), *Sox9* (post-hoc p = 0.016) (Figure 2N) but not *Col10a1* (Figure 2O) in the GFP-TDSC-Scx compared to those in the GFP-TDSC-Mock. There was also significant higher expression of bone-related markers including *Runx2* (post-hoc p = 0.047) (Figure 2P) but not *Spp1* (Figure 2Q) and *Bglap* (Figure 2R) in the GFP-TDSC-Scx compared to that in the GFP-TDSC-Mock.

### Effect of GFP-TDSC-Scx on Tendon Healing

#### Gross observation

We transplanted GFP-TDSC-Scx, GFP-TDSC-Mock or scaffold only to the patellar tendon window wound. There was no observable difference in lameness among different groups. The knees were less swollen in the GFP-TDSC-Mock and GFP-TDSC-Scx groups compared to those in the Scaffold-only group. There was reduced movement of the animals in the first few days and the animals resumed normal activity level afterwards.

#### *Ex vivo* fluorescent imaging

**Figure 3:**
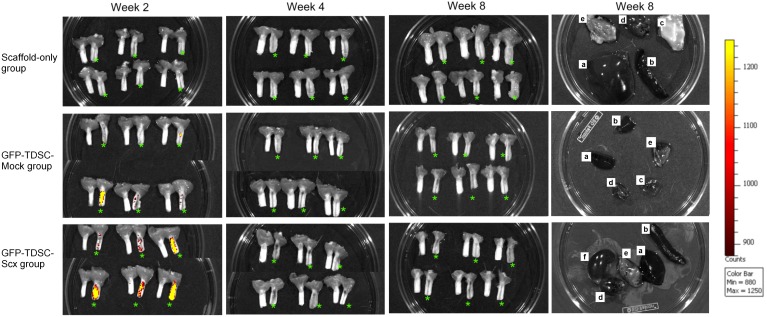
GFP signal of the transplanted GFP-TDSC-Mock and GFP-TDSC-Scx in the window wound. The background signal in the Scaffold-only group at week 2, 4, and 8 was also shown as reference. n = 6 per group per time point; “*” indicates the injured patellar tendon while the adjacent unlabeled one is the intact contralateral patellar tendon of each rat. The fluorescent images of other organs including (a) liver, (b) spleen, (c) muscle, (d) heart, (e) lung and (f) kidney at week 8 were also shown. The transplanted GFP-TDSCs could be observed in the window wound at week 2 but not at week 4 and week 8 in both cell groups. No fluorescent signal was detected in the Scaffold-only group and in other organs.

We tracked the fate of the transplanted cells in the patellar tendon window wound. *Ex vivo* fluorescent imaging showed the presence of transplanted GFP-TDSCs at week 2 but not at week 4 and week 8 in both the GFP-TDSC-Mock and GFP-TDSC-Scx groups (Figure 3). Higher signal intensity indicating more GFP-positive cells was observed in the window wound in the GFP-TDSC-Scx group compared to that in the GFP-TDSC-Mock group at week 2 (Figure 3). No fluorescent signal was detected in the Scaffold-only group and in the other organs including heart, liver, muscle, spleen, lung and kidney at all the time points (Figure 3), suggesting that the transplanted cells did not migrate to the other organs.

#### Histology

**Figure 4:**
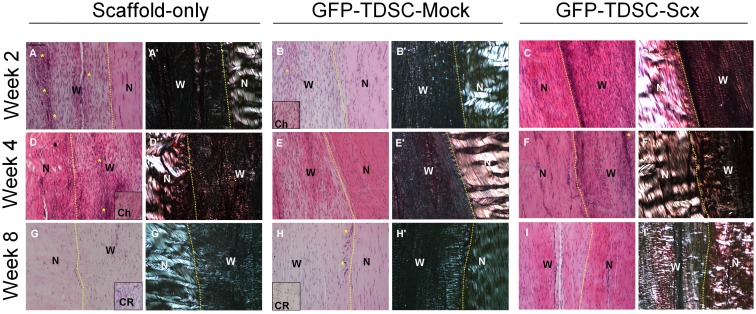
Histology of the window wound after GFP-TDSC-Scx transplantation. Representative photomicrographs showing the histology (A–I) and the corresponding polarized image (A’–I’) of the window defect in the Scaffold-only group (A, A’, D, D’, G, G’), GFP-TDSC-Mock group (B, B’, E, E’, H, H’) and GFP-TDSC-Scx group (C, C’, F, F’, I, I’) at week 2 (A–C, A’–C’), week 4 (D–F, D’-F’) and week 8 (G–I, G’–I’) after injury. Scale bar: 100 µm; Stain: haematoxylin and eosin; W: wound; N: adjacent normal tendon; Ch: chondrocyte-like cells; CR: ossified region; *: vascular features. Better healing with improvement in fiber arrangement and lower vascularity was observed in the GFP-TDSC-Scx group compared to that in the GFP-Mock group which in turns was better than that in the Scaffold-only group.

We assessed the healing in the window wound by histology and scoring. The scoring table and the representative photomicrographs were shown in Table 2 and Figure 4, respectively.

**Table 2:** Histological scoring of different groups at week 2, week 4 and week 8.

	Fiber arrangement(%)^1^	Cellularity(%)^2^	Cell alignment(%)^3^	Cell rounding(%)^4^	Vascularity(no.)^5^	Fiberstructure (%)^6^	Hyaline degeneration(%)^7^	Inflammation(%)^8^	Ossification(%)^9^	Total score^10^
**Week 2**										
Scaffold-only group	2.5 (1–3)	2.5 (0–3)	2 (1–3)	2 (2–2)	2.5 (1–3)	1 (1–1)	0 (0–0)	2 (2–2)	0 (0–0)	14 (11–15)
GFP-TDSC-Mock group	2 (1–2)	2.5 (2–3)	2 (1–2)	2.5 (2–3)	1 (1–3)	1 (1–2)	0 (0–1)	2 (2–3)	0 (0–0)	14 (10–19)
GFP-TDSC-Scx group	1 (0–2)^a^	2 (2–3)	2 (1–2)	2 (1–3)	1 (0–1)^a^	1 (1–2)	0 (0–0)	2 (1–3)	0 (0–0)	11 (9–13)^a^
**Week 4**										
Scaffold-only group	1 (1–3)	2 (1–3)	1 (0–2)	2 (1–2)	2.5 (0–3)	1.5 (1–2)	0 (0–2)	1.5 (1–2)^x^	0 (0–0)	12 (7–16)
GFP-TDSC-Mock group	0.5 (0–1)^a^	3 (2–3)	1.5 (0–2)	1.5 (1–2)^x^	1 (1–1)	1.5 (1–2)	0 (0–0)	0.5 (0–2)^x^	0 (0–0)	9 (6–13)
GFP-TDSC-Scx group	0 (0–2)^a^	2 (1–3)	1 (0–2)	1 (1–2)^x^	1 (0–1)	1.5 (1–2)	0 (0–0)	0.5 (0–1)^x^	0 (0–0)	8 (5–9)^x^
**Week 8**										
Scaffold-only group	1 (0–3)	0 (0–2)^x^ **^,^** y	1 (0–2)	1 (0–2)	1 (0–3)	2 (0–3)	0 (0–2)	1 (0–2)^x^	0 (0–1)	8 (2–16)
GFP-TDSC-Mock group	0.5 (0–3)	2 (2–2)^a^ **^,^** x **^,^** y	0.5 (0–3)	1 (1–2)^x^	1 (1–2)	1.5 (0–2)	0 (0–1)	1 (0–2)^x^	0 (0–1)	7.5 (5–17)
GFP-TDSC-Scx group	0 (0–1)^x^	1 (1–2)^b^ **^,^** x **^,^** y	0.5 (0–2)	1 (1–1)^x^	1 (1–2)	0.5 (0–1)^x^ **^,^** y	0 (0–0)	0 (0–1)^a^ **^,^** b **^,^** x	0 (0–0)	5.5 (3–8)^x^

median (range).

Better total score was observed in the GFP-TDSC-Scx group compared to that in the GFP-Mock group which in turns was better than that in the Scaffold-only group. The total score was significantly better in the GFP-TDSC-Scx group compared to that in the Scaffold-only group at week 2. The better total score in the GFP-TDSC-Scx group was mainly contributed by the improvement in fiber arrangement, vascularity, fiber structure and degree of inflammation.

**Footnote:**

1 **Fiber arrangement:** Only fibers with typical collagen birefringence of tendon are regarded as good; reported as the areal percentage of poor fibers.

**0:** *0%*; **1:** *≤10%*; **2:** *≤30%*; **3:** *>30%.*

2 **Cellularity:** calculated as the percentage increase in cellularity compared to normal tendon.

**0:** *normal (0%)*; **1:** *≤50%*; **2:** *≤150%*; **3:** *>150%.*

3 **Cell alignment:** calculated as the areal percentage of cells not aligned along the direction of tensile load in tendon.

**0:** *≤10%*; **1:** *≤20%*; **2:** *≤50%*; **3:** *>50%.*

4 **Cell rounding:** calculated as the areal percentage of non-spindle-shaped cells.

**0:** *0%*; **1:** *≤10%*; **2:** *≤30%*; **3:** *>30%.*

5 **Vascularity:** calculated as the number of vascular features.

**0:** *0*; **1:** *≤5*; **2:** *≤10*; **3:** *>10.*

6 **Fiber structure:** calculated as the areal percentage with fibers that were separated and wavy.

**0:** *straight and tightly-packed (0%)*; **1:** *slight waviness and fiber separation (≤10%)*; **2:** *moderate waviness and fiber separation (≤30%)*; **3:** *severe waviness and fiber separation (>30%).*

7 **Hyaline degeneration:** calculated as the areal percentage with chondrocyte-like cells or ossification or degeneration.

**0:** *0%*; **1:** *≤10%*; **2:** *≤30%*; **3:** *>30%.*

8 **Inflammation:** calculated as the areal percentage with inflammatory cells characterized as very small cells with small round nuclei and scanty cytoplasm.

**0:** *0%*; **1:** *≤2%*; **2:** *≤10%*; **3:** *>10%.*

9 **Ossification:** calculated as the areal percentage with ectopic ossification.

**0:** *0%*; **1:** *≤15%*; **2:** *≤30%*; **3:** *>30%.*

10 **Total score:** calculated by summing up the scores of item (a) to (i).

a post-hoc p≤0.050 versus Scaffold-only group.

b post-hoc p≤0.050 versus GFP-TDSC-Mock group.

x post-hoc p≤0.050 versus week 2.

y post-hoc p≤0.050 versus week 4.

Italics un-bold symbol means marginally insignificant.

Overall, there was improvement in healing in all groups with time. In particular, there was significant reduction of total score in the GFP-TDSC-Scx group at week 4 (post-hoc p = 0.007) and week 8 (post-hoc p = 0.004) compared to that at week 2 (Table 2, Figure 4). Better healing (as defined by an improvement in total score) was observed in the GFP-TDSC-Scx group compared to that in the GFP-Mock group and Scaffold-only group at week 2, 4 and 8, but with the only statistically significant improvement being detected at week 2 compared to the Scaffold-only group (post-hoc p = 0.019) (Table 2, Figure 4).

The overall improvement in healing in the GFP-TDSC-Scx group compared to the other two groups was contributed by the improvement in fiber arrangement in the GFP-TDSC-Scx group compared to that in the Scaffold-only group at both week 2 (post-hoc p = 0.018) and week 4 (post-hoc p = 0.022) (Table 2, Figure 4 A’ versus C’, D’ versus F’). The vascularity was also lower in the GFP-TDSC-Scx group compared to that in the Scaffold-only group at week 2 (post-hoc p = 0.007) (Table 2, Figure 4A, *). The degree of inflammation was also significantly lower in the GFP-TDSC-Scx group compared to that in both the Scaffold-only group (post-hoc p = 0.041) and the GFP-TDSC-Mock group (post-hoc p = 0.026) at week 8 (Table 2). The GFP-TDSC-Scx group showed lower cellularity compared to that in the GFP-TDSC-Mock group at week 8 (post-hoc p = 0.019) (Table 2). There was also significant time-dependent improvement in fiber arrangement (week 2 versus week 8: post-hoc p = 0.026) and fiber structure (week 2 versus week 8: post-hoc p = 0.019; week 2 versus week 4: post-hoc p = 0.019) in the GFP-TDSC-Scx group but not in the other two groups (Table 2). One sample in the GFP-TDSC-Mock group showed chondrocyte-like cells at week 2 (Figure 4B, insert). One sample in the Scaffold-only group showed chondrocyte-like cells at week 4 (Figure 4D, insert). At week 8, two samples in the Scaffold-only group (Figure 4G) and one sample in the GFP-TDSC-Mock group showed chondrocyte-like cells (Figure 4H). Similar to the GFP-TDSC-Scx group, the GFP-TDSC-Mock group also showed better fiber arrangement compared to the Scaffold-only group at week 4 (post-hoc p = 0.054) (Table 2). However, the TDSC-Mock group showed higher cellularity compared to the Scaffold-only group at week 8 (post-hoc p = 0.01) (Table 2). Ossification was observed in the GFP-TDSC-Mock group and the Scaffold-only group at week 8 (Figure 4 G, H, insert) in histology.

#### vivaCT imaging

**Figure 5:**
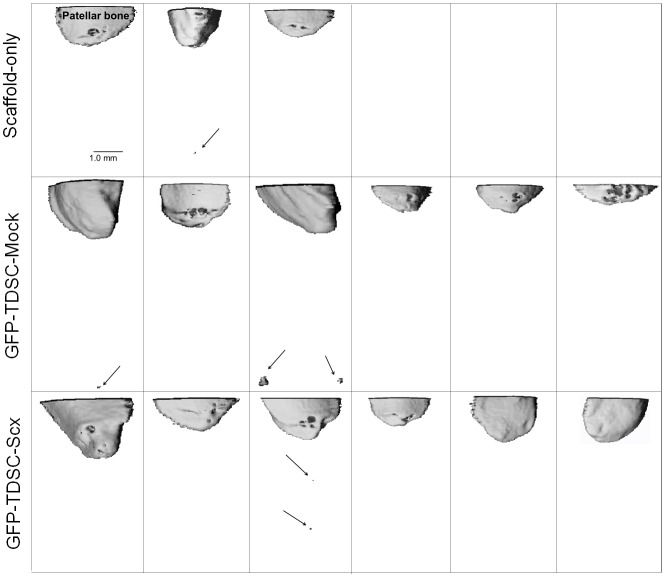
vivaCT images of ectopic mineralized tissue inside the patellar tendon at week 8 after injury. Scale bar = 1 mm; Arrows: ectopic mineralized tissue. Small mineralized tissue was observed inside the patellar tendons and there was no difference among different groups.

We assessed whether there is any difference in ectopic mineralization in tendon after injury in different groups by vivaCT imaging. Mineralized tissues were detected inside the patellar tendon in 1/3, 2/6 and 1/6 samples in the Scaffold-only group, GFP-TDSC-Mock group and GFP-TDSC-Scx group, respectively (Figure 5). The mineralized tissue was small and there was no difference among different groups (Figure 5).

#### Biomechanical test

**Figure 6:**
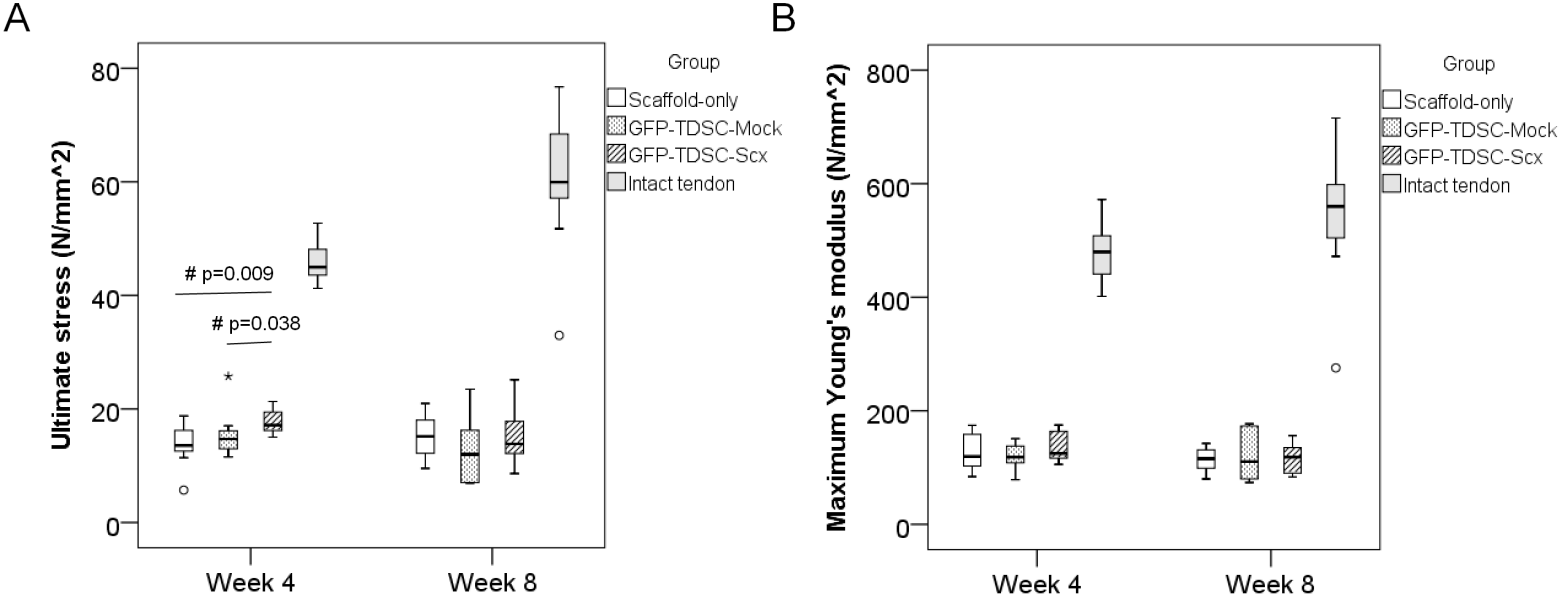
Biomechanical properties of repaired tissue after GFP-TDSC-Scx transplantation. Boxplots showing (A) the ultimate stress and (B) the maximum Young’s modulus in the Scaffold-only group, GFP-TDSC-Mock group, GFP-TDSC-Scx group and the intact tendon control group at week 4 and week 8 after injury. #p≤0.05 in post-hoc comparison; “o” and “*” represent outlier and extreme value of the dataset, respectively. The ultimate stress in the GFP-TDSC-Scx group was significantly higher compared to those in the Scaffold-only group and the GFP-TDSC-Mock group at week 4.

The quality of the repaired tissue in different groups was assessed by biomechanical test. The ultimate stress in the GFP-TDSC-Scx group was significantly higher compared to that in the Scaffold-only group (overall p = 0.019; post-hoc p = 0.009) and the GFP-TDSC-Mock group (post-hoc p = 0.038) at week 4 (Figure 6A). There was no difference in ultimate stress among different groups at week 8 (Figure 6A). There were also no difference in the maximum Young’s modulus among different groups at week 4 and week 8 (Figure 6B). About half of the samples failed at the mid-substance at both week 4 and week 8. There was no difference in the failure mode among different groups at different time points (Table 3).

**Table 3:** The failure mode of healing tendon tissue in different groups at week 4 and week 8.

	Tibia	Patellar	Mid-substance	Total
**Week 4**				
Scaffold-only group	**4**	**1**	**5**	**10**
GFP-TDSC-Mock group	**2**	**2**	**6**	**10**
GFP-TDSC-Scx group	**2**	**1**	**7**	**10**
**Week 8**				
Scaffold-only group	**0**	**3**	**5**	**8**
GFP-TDSC-Mock group	**1**	**1**	**3**	**5**
GFP-TDSC-Scx group	**2**	**1**	**4**	**7**

There was no difference in the failure mode among different groups at different time points.

#### Immunohistochemical staining of collagen type I

**Figure 7:**
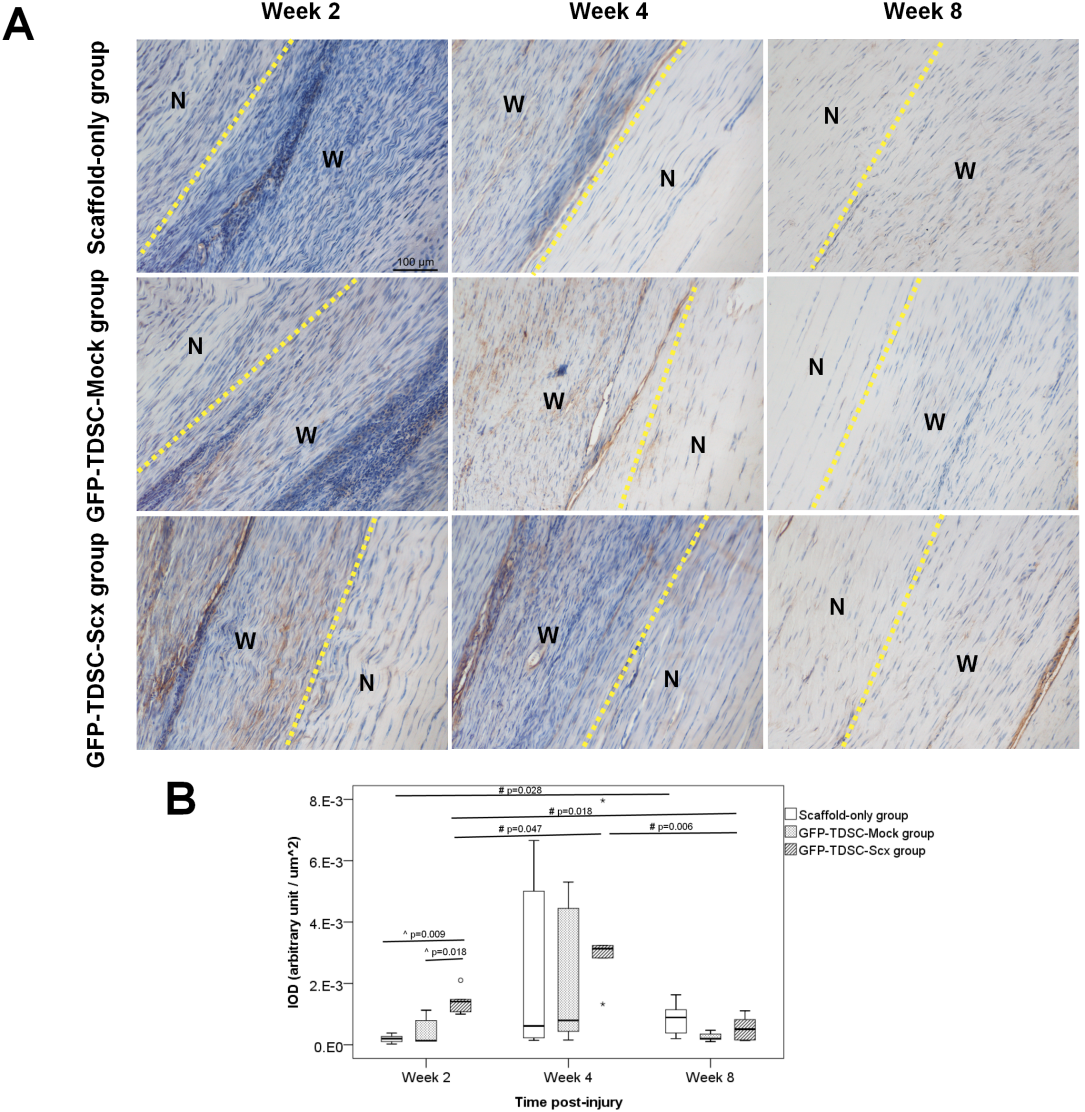
Immunohistochemical staining of collagen type I in the window wound. (A) Representative photomicrographs; Scale bar: 100 µm; W: wound; N: adjacent normal tendon; (B) Boxplot showing integrated optical density (IOD) of collagen type I in the window wound. “o” and “*” represent outlier and extreme value of the dataset, respectively. ^∧^p≤0.05 compared to the GFP-TDSC-Scx group; ^#^post-hoc p≤0.05 compared to different time points in the same treatment group; n = 5–6/group/time point. There was significant higher expression of collagen type I in the window wound in the GFP-TDSC-Scx group compared to the other two groups at week 2.

We examined the potential healing mechanism of GFP-TDSC-Scx by studying the expression of collagen type I in the window wound. Similar to our previous findings, the expression of collagen type I increased from week 2 to week 4 and then reduced slightly at week 8 in all three groups (overall p = 0.012, 0.026 and 0.001 for Scaffold-only group, GFP-TDSC-Mock group and GFP-TDSC-Scx group, respectively) (Figure 7). There was significant higher expression of collagen type I in the window wound in the GFP-TDSC-Scx group compared to those in the Scaffold-only group (overall p = 0.014; post-hoc p = 0.009) and the GFP-TDSC-Mock group (post-hoc p = 0.018) at week 2 (Figure 7).

## Discussion

Unlike BMSCs, non-transduced TDSCs showed high constitutive expression of *Scx* [32]. The transduction of lentiviral vector containing the *Scx* gene into TDSCs increased *Scx* expression while the transduction of empty lentiviral vector into TDSCs reduced *Scx* expression compared to the non-transduced TDSCs. TDSC-Mock and TDSC-Scx, while tested at the same passage as the non-transduced TDSCs, were actually older cells. Our previous study reported reduced expression of tendon-related markers including *Scx*, in TDSCs with *in vitro* cell passaging [23]. Zhou *et al.* [33] also reported that tendon-derived stem/progenitor cells isolated from aged rats (24–26 months) showed lower mRNA expression of *Scx* and other tendon-related markers compared to similar cells isolated from young rats (3–4 months). This was not an issue in Alberton *et al.* [19] ’s study because they used immortalized BMSCs. However, the use of immortalized cells for tissue engineering raised the concern of tumor formation and hence is an issue that needs to be considered and evaluated carefully prior to clinical application.

Our published results showed that TDSCs have low immunogenicity and hence are suitable for allogeneic transplantation in tissue engineering [24], [25]. TDSCs have an advantage over BMSCs as a cell source for musculoskeletal tissue repair as they proliferate faster, exhibit higher clonogenicity and multi-lineage differentiation potential compared to BMSCs [32]. In fact, we had transduced un-immortalized paired GFP rat BMSCs with *Scx* using the same lentiviral system but the cells failed to grow after blasticidin selection (results not shown). This might be because BMSCs aged and lost multi-lineage differentiation potential more rapidly compared to TDSCs. This was supported by a previous study which showed that BMSCs isolated from SD rats aged after 2 passages with reduced osteogenic differentiation potential [34]. Further study is required to confirm this speculation.

While it is known that TDSCs express higher level of tenogenic markers including *Scx* compared to paired BMSCs, TDSCs also exhibit higher osteogenic, chondrogenic and adipogenic differentiation potential compared to BMSCs [32] and hence might also have the chance of erroneous differentiation after transplantation. Also, while the expression of *Scx* in TDSCs is higher than that in BMSCs [32], this is relative to BMSCs only. Lentiviral transduction of *Scx* further increased *Scx* expression in TDSC as shown in this study. It is also not clear if increasing the tenogenic activity of TDSCs by *Scx* transduction prior to transplantation would enhance the production of appropriate tendon matrix and promote better tendon repair compared to the mock group. These uncertainties justified the conduct of this study.

Gulotta *et al.* [20] used an adenoviral system for the transduction of *Scx* into BMSCs. No antibiotic was used for cell selection. However, not all cells were transduced with *Scx* gene with the use of an adenoviral system. As the transplanted cells were seen in the window wound at week 2 but not at week 4 after transplantation, whether transient transduction would yield similar or better results compared to the stable transduction of *Scx* in the promotion of tendon repair needs further research.

While the transduction of *Scx* into GFP-TDSCs seemed to consistently increase the expressions of most tendon-related and cartilage-related markers, its effect on the expressions of bone-related markers was inconclusive. GFP-TDSC-Scx showed significantly higher expression of *Col1a1*, consistent with the literature about the role of Scx in regulating the expression of collagen type I [2], [35], [36]. Alberton *et al.* [19] showed higher secretion of collagen type I in the *Scx*-transduced immortalized BMSCs compared to that in the mock-transduced cells, but there was no difference in the mRNA level of *COL1A1* between the two groups. A previous study showed that *Scx* was essential for the expression of *Tnmd* [37] and sequential expressions of *Scx* and *Tnmd* were observed after treatment of BMSCs with BMP-12 [38]. Our result was different from Alberton *et al*. [19] ’s report. The transduction of *Scx* did not increase the mRNA expression of *Tnmd* in GFP-TDSCs. This might be due to the difference in cell source and the use of cells with limited life span in this study. We tested more tendon-related markers including *Thbs4*, *Tnc*, *Eln* and *Epha4*, *Eya1*, *Six1* and *Six2* in this study. Our result was different from Alberton *et al.* [19] ’s study regarding the effect of *Scx* transduction on the expression of cartilage-related markers. They reported that *SCX* transduction reduced the expression of *SOX9* in the immortalized BMSCs compared to that in the mock control. There were no difference in the mRNA expression of *BGN* and no detectable expression of *ACAN* between the transduced and non-transduced cells in Alberton *et al.* [19]’s report. However, we reported higher expressions of cartilage-related markers including *Col2a1*, *Sox9*, *Acan* and *Bgn* in the GFP-TDSC-Scx compared to those in the GFP-TDSC-Mock. In fact, there were also studies supporting Scx as an important regulator of chondrogenesis in addition to tenogenesis [39]–[44] and Scx alone might not be sufficient to commit tenogenic differentiation [45]. The fate of GFP-TDSC-Scx should be further confirmed by the type of neo-tissue formed after transplantation using the nude mouse model.

Gulotta *et al.* reported that the transplantation of BMSC-Scx [20], but not BMSCs [21], promote tendon to bone junction repair in a rotator cuff injury model, suggesting that Scx is important for tendon to bone junction healing. We reported that the transplantation of GFP-TDSC-Scx histologically and biomechanically promoted better tendon repair compared to the GFP-TDSC-Mock and Scaffold-only groups at the early stage after injury. A previous study reported that the transplantation of BMSCs at high concentration induced ectopic bone formation during tendon repair [8], [11]–[12]. Our results showed that the transplantation of GFP-TDSC-Mock and GFP-TDSC-Scx did not increase the risk of ectopic chondro-ossification in the patellar tendon window injury model, a model which was shown to show ectopic chondrocyte-like cells and ossified deposits in some samples at the later stage of tendon healing [4]. Our results hence suggested that the transplantation of TDSCs or TDSC-Scx for tendon repair was safe. Our previous study showed that the transplantation of early passage of TDSCs to the window wound promoted tendon repair up to week 4 [17]. No significant improvement was observed at the later time points after transplantation of GFP-TDSC-Scx in this study. Unlike the previous study [17], the transplantation of GFP-TDSC-Mock did not significantly promote tendon repair. Both might be due to the use of older cells with lower tenogenic differentiation potential. Comparing the biomechanical test data of these two studies, the biomechanical properties of the repair tissue in the GFP-TDSC-Scx group with cells at the later passage were at least as good as the non-transduced GFP-TDSC group with cells at the early passage in the previous study [17]. The transduction of *Scx* might “rescue” or “improve” later passaged cells but they might not be better than using non-transduced cells at earlier passages which would be easier and less regulated for clinical use. Further research is required to confirm this.

GFP-TDSCs could not be detected in the window wound at week 4 after transplantation. This was in line with the removal of excess cells during tendon remodeling [46]. Similar result was observed with the transplantation of allogeneic BMSCs [47]–[48]. The authors also attributed the decrease in the number of transplanted cells to tendon remodeling [48]. We hypothesized that GFP-TDSCs might promote tendon repair by modulating the early inflammatory response and producing trophic factors [49]–[51]. We showed that the transplantation of GFP-TDSC-Scx promoted the expression of collagen type I in the window wound, consistent with the role of Scx as a key regulator of collagen type I mRNA transcription [2]. Our unpublished results also showed that GFP-TDSC expressed bFGF *in vitro* which might promote tendon repair [52]. Further research is required to confirm this speculation. The earlier down-regulation of vascularity and the better arrangement of collagen fibers might also contribute to better tendon repair in the GFP-TDSC-Scx group but the mechanisms involved are unclear at the moment. There were trends of reduced expression of collagen type I in the window wound at week 8 compared to that at week 4 in all three groups. This temporal change of collagen type I expression was consistent with our previous findings [4]. In that study, we showed that the expression of collagen type I in the window wound remained higher than the intact control up to week 12 after injury despite the decrease at week 4 compared to that at week 2 [4].

## Conclusions

In conclusion, GFP-TDSC-Scx consistently showed higher expressions of most of the tendon-related and cartilage-related markers compared to the GFP-TDSC-Mock. However, the effect of Scx transduction on the expressions of bone-related markers was inconclusive. The GFP-TDSC-Scx group only statistically significantly improved tendon repair histologically and biomechanically compared to the Scaffold-only group and the GFP-TDSC-Mock group at the early stage of tendon repair. GFP-TDSC-Scx group might promote early tendon repair by increasing the expression of collagen type I in the window wound.

## Supporting Information

Supporting Information S1 **Supplementary protocol for the isolation and culture of TDSCs.**
(DOCX)
