# Supplementary material for: Scx-Transduced Tendon-Derived Stem Cells (TDSCs) Promoted Better Tendon Repair Compared to Mock-Transduced Cells in a Rat Patellar Tendon Window Injury Model
Source: PLoS One. 2014 May 15;9(5):e97453. doi: 10.1371/journal.pone.0097453 (PMC4022525; doi:10.1371/journal.pone.0097453)
Supplement: Supporting Information S1 — Supplementary protocol for the isolation and culture of TDSCs. (DOCX) [file pone.0097453.s001.docx]

**Supporting information S1.** Supplementary protocols for the isolation and culture of TDSCs

The mid-substance of patellar tendon was excised from healthy rats overdosed with 2.5% sodium phenobarbital i.p (1.0ml/400g). Care was taken that only the mid-substance of patellar tendon tissue, but not the tissue at the bone-tendon junction, was collected. Peritendinous connective tissue was carefully removed and the tissue was stored in sterile phosphate-buffered saline (PBS). The tissue was minced, digested with type I collagenase (3mg/ml; Sigma-Aldrich, St Louis, MO, USA) and passed through a 70μm cell strainer (Becton Dickinson, Franklin Lakes, NJ, USA) to yield single-cell suspension. The released cells were washed in PBS and resuspended in low glucose Dulbecco’s Modified Eagle Medium (LG-DMEM) (Gibco BRL; Life Technologies, Invitrogen, Carlsbad, CA, USA), 10% fetal bovine serum (FBS), 50µg/ml penicillin, 50µg/ml streptomycin and 100µg/ml neomycin (all from Invitrogen, Carlsbad, CA, USA). The isolated nucleated cells were plated at an optimized low density (500 cells/cm^2^) for the isolation of TDSCs from rat patellar tendon and cultured at 37°C, 5% CO_2_ to form colonies. At day 7-10, the adherent cells were trypsinized and mixed together as passage 0 (P0). TDSCs were subcultured when they reached 80-90% confluence. Medium was changed every three days.
